# A Case of Pyoderma Gangrenosum in a 40-Year-Old Male Patient: A Challenging Diagnosis

**DOI:** 10.7759/cureus.79051

**Published:** 2025-02-15

**Authors:** Jatin Goyal, Alexandra Goldman, Nicole Cardona

**Affiliations:** 1 Internal Medicine, Florida International University, Herbert Wertheim College of Medicine, Miami, USA; 2 Internal Medicine, Baptist Health South Florida, Miami, USA

**Keywords:** cellulitis, chronic leg ulcer, corticosteroids, immunosuppressive therapy, neutrophilic dermatosis, peripheral vascular disease (pvd), pyoderma gangrenosum

## Abstract

Pyoderma gangrenosum (PG) is a rare, immune-mediated neutrophilic dermatosis, presenting with painful ulcerative skin lesions. These lesions often start as pustules on an erythematous base, progressing to large ulcers with purulent edges. Diagnosing PG can be challenging, as it lacks definitive tests and requires exclusion of other conditions, including infections and vascular diseases. PG is frequently associated with systemic autoimmune diseases such as inflammatory bowel disease, rheumatoid arthritis, and monoclonal gammopathies. This report describes a case of a 40-year-old Hispanic male patient with recurrent, painful lesions on his lower extremities. Initially misdiagnosed as cellulitis, the patient had a complex history of peripheral vascular disease, which added to the diagnostic difficulty. The patient's history of recurrent ulcerations, persistent post-inflammatory hyperpigmentation, and rapid response to corticosteroids led to a clinical diagnosis of PG. This case highlights the need for careful differential diagnosis in chronic, non-healing lesions, emphasizing that PG should be considered, particularly in atypical presentations or in patients without common systemic associations. Early recognition and immunosuppressive therapy are crucial to avoid misdiagnosis and improve patient outcomes.

## Introduction

Pyoderma gangrenosum (PG) is a rare, immune-mediated condition characterized by painful, ulcerative skin lesions. PG is classified as a neutrophilic dermatosis, due to the abundance of neutrophils found in the affected tissue. However, this neutrophilic predominance is not due to an infection, but instead an auto-inflammatory response. Clinically, PG lesions often begin as small pustules on an erythematous base, which gradually evolves into larger, ulcerative lesions. These ulcers can vary in depth, ranging from shallow to deep, and are typically accompanied by intense pain. The edges of the ulcerations are characteristically purulent and may present with a surrounding erythematous or violaceous border. These features, while common in PG, are not exclusive to the condition, making diagnosis challenging [1,2]. Without definitive diagnostic tests, the diagnosis often relies on clinical assessment and the exclusion of other conditions, such as bacterial, viral, fungal, and parasitic infections, as well as other neutrophilic dermatoses, including Sweet syndrome, insect bites, and vascular conditions like venous insufficiency or peripheral arterial disease (PAD) [3,4].

Though PG can be idiopathic, it is strongly associated with systemic diseases, particularly inflammatory bowel diseases (IBD) such as ulcerative colitis and Crohn’s disease. PG also has significant associations with autoimmune conditions like rheumatoid arthritis and monoclonal gammopathies [1,4]. Recent studies suggest that PG is a pro-inflammatory condition characterized by elevated levels of various interleukins and cytokines that play a key role in immune regulation. These interleukins promote both the recruitment and activation of neutrophils, leading to the development of the characteristic lesions [5-7]. In addition, genetic factors have been implicated in PG, with certain genetic markers associated with an increased predisposition to the condition [8].

Despite these advances in understanding PG, its diagnosis remains complex, particularly in cases where the presentation is atypical or there is an absence of commonly associated systemic conditions. The variability in clinical presentation and the frequent misdiagnosis of PG as infectious or vascular pathology contribute to delays in treatment and suboptimal patient outcomes. Here, we present the case of a 40-year-old Hispanic man in South Florida who developed recurrent, painful, erythematous lesions on his anterior tibia bilaterally. His presentation was atypical, lacking clear features of PG and without a known history of associated comorbidities, highlighting the diagnostic challenges associated with PG.

## Case presentation

A 40-year-old Hispanic man with a past medical history of chronic lesions on both lower extremities, varicose veins, and familial hypertriglyceridemia associated with peripheral arterial disease presented to the emergency department with worsening pain, swelling, and redness in his right leg. About a week before his visit, the patient experienced systemic symptoms, including fever, chills, and body aches, which led him to stay in bed for approximately 48 hours. Once his systemic symptoms subsided and he resumed walking, he noticed increasing redness, swelling, and pain in his right leg, which radiated toward his groin. The patient had undergone an ultrasound of the right lower extremity just a few days prior to this visit, which ruled out deep vein thrombosis. Despite this, the symptoms persisted and progressively worsened, prompting him to go to the emergency room. According to the patient, these symptoms and lesions were different from his previous episode of PAD, which required femoral artery stenting. However, he recalled having similar lesions in the past, though they were not accompanied by such severe systemic symptoms or pain. The patient denied any chest pain, shortness of breath, fever, chills, nausea, vomiting, or diarrhea at the time of presentation.

**Figure 1:**
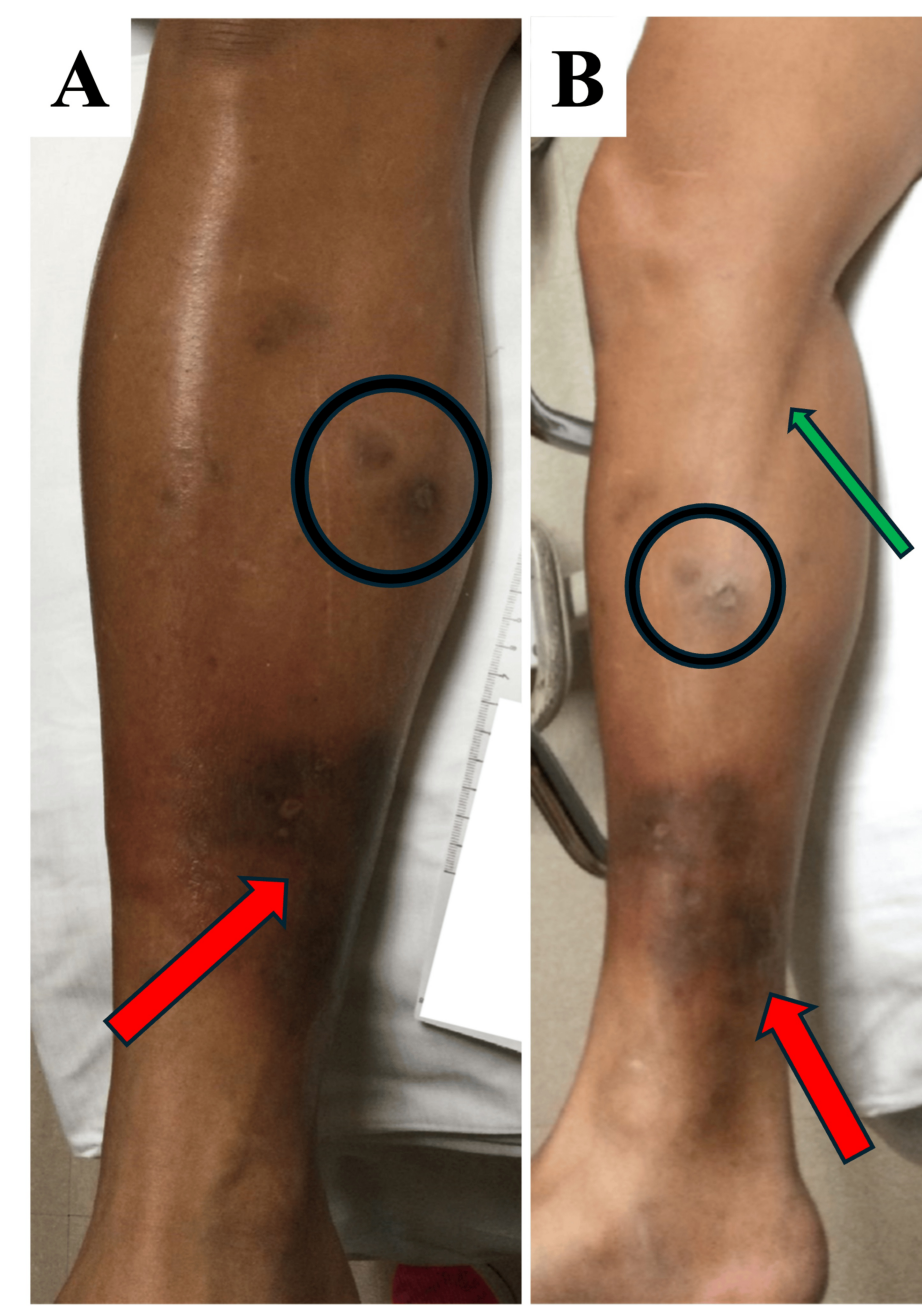
Lesions on the right lower extremity A) Right lower extremity with an anterior view. B) Right lower extremity with a medial view. Red arrow: current lesions; black circle: previously healed lesions, with hyperpigmented scarring; green arrow: lymphangitis.

On physical examination, the medial aspect of the patient’s right lower extremity displayed a region of hyperpigmentation with surrounding erythema, warmth, and tenderness (Figure 1). The erythema extended in a linear pattern along the medial thigh, and the area was notably painful to touch. The left leg, while showing signs of hyperpigmentation from past ulcerations, did not exhibit erythema or other acute inflammatory signs. Initial laboratory results revealed a white blood cell count of 11.6 x 10^9^/L, a platelet count of 456 x 10^9^/L, an erythrocyte sedimentation rate (ESR) of 23 mm/hour, and an elevated lactic acid level of 3.7 mmol/L, which later decreased to 1.7 mmol/L (Table 1). Imaging, including a right tibia-fibula X-ray and a computed tomography (CT) scan of the right lower extremity, revealed no fractures, osteomyelitis, soft tissue emphysema, abscesses, or foreign bodies. Blood cultures were sent at this time.

**Table 1:** Laboratory results during hospitalization ESR: Erythrocyte sedimentation rate, CRP: C-reactive protein

Laboratory Tests	Hospital Day					
	1	2	3	4	5	Reference Range
White Blood Cells (K/uL)	11.6	14.0	-	9.92	9.64	3.40-11.0
Red Blood Cells (M/uL)	5.36	5.36	-	5.16	5.16	4.00-5.70
Hemoglobin (g/dL)	15.9	15.5	-	14.7	15.0	13.0-17.2
Hematocrit (%)	44.7	44.7	-	43.7	44.1	38.0-50.0
Platelet Count (K/uL)	456	346	-	378	393	130-360
ESR (mm/hr)	23.0	-	29.0	29.0	-	0.00-20.0
Lactic Acid (mmol/L)	3.70	-	-	1.70	-	0.50-2.20
CRP (mg/L)	40.0	-	77.0	88.0	-	0.00-10.0

Given these findings, the working diagnosis was right lower extremity cellulitis, and the patient was started on intravenous vancomycin and cefepime to treat a presumed bacterial infection. Lymphangitis, as indicated by the linear erythema extending up the thigh, was also suspected. The vascular surgery team was consulted but did not recommend any surgical intervention.

By the following day, the patient’s condition had not improved significantly. His white blood cell count increased to 14.0 x 10^9^/L, and his C-reactive protein (CRP) had risen to 77 mg/L. A repeat X-ray and CT scan did not show any signs of abscess, acute osteomyelitis, or significant joint effusion. Infectious disease (ID) specialists were consulted to review the case. As per ID, due to the recurrent nature and appearance of these lesions and the persistent post-inflammatory hyperpigmentation, there was greater suspicion of a non-infectious process, possibly PG. The ID specialist recommended discontinuation of the vancomycin infusion and initiation of an infusion with cefazolin 2g IV every eight hours to address any superimposed infection. The patient was also started on a treatment of corticosteroids (prednisone 40 mg IV daily) to address the suspected PG. Inflammatory markers were to be monitored, and dermatology was consulted for further evaluation.

On the third day of admission, the corticosteroid treatment and antibiotics were continued due to the patient's current clinical improvement. Further diagnostic tests, including an antinuclear antibody panel, rheumatoid factor, anti-citrullinated protein antibodies, and complement levels, were ordered (Table 2). Inflammatory markers remained elevated, with the ESR rising to 29 mm/hour, and CRP increasing to 80 mg/L as noted in Table 1. On day four of hospitalization, the blood cultures were reported as negative. Dermatology evaluated the patient and concurred that the most likely diagnosis at this time is PG. Their suspicion was based on the physical presentation of these recurrent lesions as well as the significant improvement once the corticosteroids were started. Though a biopsy of the lesions could confirm the diagnosis, dermatology's recommendation was to avoid a biopsy at this time in order to prevent further worsening of the lesions. Therefore, the patient was started on a prolonged 18-day prednisone taper regimen.

**Table 2:** Additional diagnostic tests ordered

Diagnostic Tests	Result	Reference Range
Antinuclear Antibody	Negative	Negative
Rheumatoid Factor	Negative	Negative
Complement CH50 (U/mL)	>60	>41
Anti-Cyclic Citrullinated Peptide Antibody (U/mL)	<20	<20

By day five, the patient had shown significant clinical improvement. Erythema, swelling, and pain in his right leg had markedly decreased with steroid therapy. His inflammatory markers stabilized, and he reported feeling better. The ID team recommended completing two additional days of cefazolin as an outpatient. He was discharged with instructions to continue the steroid taper and follow up with dermatology. The final diagnosis was likely PG, with his rapid clinical response to corticosteroids supporting this conclusion.

## Discussion

Due to the lack of definitive diagnostic testing, PG is often misdiagnosed, commonly mistaken for conditions like PAD or polyarteritis nodosa [9]. While this patient has a history of PAD, the recurrence of lesions after revascularization suggested an alternate etiology. Polyarteritis nodosa was ruled out due to the absence of hallmark features, including arthralgia, palpable purpura, abdominal pain, and livedo reticularis. Similarly, other immune-mediated conditions, such as vasculitis, Behcet’s disease, and Sweet syndrome, were deemed less likely as the patient’s lesions were more characteristic of PG [10,11].

In this case, the patient's lesions were located on the anterior tibia, which is the most common site for PG. However, the presentation did not clearly align with any of the established five subtypes: pustular, ulcerative, bullous, vegetative, or peristomal. The ulcerative and pustular forms are most frequently associated with IBD, with pustules in the ulcerative form often progressing to ulcers. Ulcerative PG is also linked to autoimmune conditions such as rheumatoid arthritis and monoclonal gammopathies. The bullous form initially presents as bullae, mainly affecting the upper body or extremities, which subsequently erode into superficial ulcers. The vegetative form is characterized by superficial ulcers with verrucous lesions but lacks the violaceous undermined borders seen in other types. Peristomal PG typically manifests as painful papules that ulcerate around stoma sites [2].

The initial presentation in this case did not immediately suggest PG. At first, the rash was more consistent with cellulitis, and when well-demarcated lesions formed, they did not ulcerate, further complicating the diagnosis. Additionally, the patient did not have an underlying history of IBD or other immune-mediated disease, which can be commonly associated with PG. These atypical features contributed to the diagnostic challenge [12]. However, the recurrence of the lesions, combined with the exclusion of alternative diagnoses, ultimately increased the suspicion for PG [13].

The standard treatment for acute PG typically involves rapid-acting immunosuppressive therapy, with corticosteroids or cyclosporine being the mainstays. In this case, corticosteroids were selected due to their rapid onset of action and physician familiarity with their use. Cyclosporine was avoided due to its potential nephrotoxic effects, which were deemed an unnecessary risk in this patient. Long-term management often includes steroid-sparing agents, allowing for a gradual taper of corticosteroids once the acute episode has resolved, as indicated by lesion stabilization or regression [1,14]. Although the final diagnosis was not confirmed by biopsy, the patient’s marked clinical improvement following IV prednisone therapy strongly supported PG as the most likely etiology. In alignment with established treatment guidelines, the patient was prescribed a prednisone taper for ongoing management and referred to dermatology for continued follow-up and maintenance therapy.

## Conclusions

This case highlights the diagnostic challenges of PG, particularly in patients with atypical presentations or without a history of associated systemic conditions like IBD. The recurrent, non-healing lesions on the patient's anterior tibia, initially suspected to be cellulitis, required careful exclusion of other differential diagnoses, including PAD and polyarteritis nodosa, before PG emerged as the most likely cause. The patient’s significant clinical improvement with corticosteroid therapy further supported this diagnosis. Clinicians should remain vigilant for the rare condition of PG, especially in patients with a history of recurrent non-infectious ulcers or lesions or in cases lacking classic clinical associations. Early recognition and timely initiation of immunosuppressive therapy can promote rapid improvement and prevent unnecessary interventions.
